# China’s new urban clusters strategy for coordinated economic growth: Evidence from the sports industry

**DOI:** 10.1371/journal.pone.0292457

**Published:** 2023-10-03

**Authors:** Lide Su, AGUDAMU, Yuqian Liu, Yang Zhang

**Affiliations:** 1 School of Humanities, Inner Mongolia University of Technology, Hohhot, China; 2 Institute of Sports and Health Industry, HEHA CAT Fitness, Changsha, China; 3 Graduate School of Social Welfare, Sungkyunkwan University, Seoul, South Korea; 4 School of Finance, Hunan University of Finance and Economics, Changsha, China; 5 Independent Person, Windermere, Florida, United States of America; Hosei University: Hosei Daigaku, JAPAN

## Abstract

In 2014, the Chinese government unveiled the New Urbanization Plan and Document No. 46, which profoundly influenced the development trajectory of the regional economy and sports industry. Using the coupling coordination model, this study aimed to assess the development progress of the sports industry and urban clusters economy. This study sampled Greater Bay Area urban clusters (GBAUC) and Yangtze River Delta urban clusters (YRDUC). The statistics covered one year after the release of the policies to date. We developed a total of 15 macro indicators to evaluate the sports industry and urban cluster economy as two distinct, yet interdependent, economic systems. Using the entropy weight method, we determined the standardized values and weights for the two systems before calculating the coupling coordination degree (D). Between 2015 and 2021, the sampled sports industry and urban clusters economy exhibited coordinated high growth across all economic metrics, with multiple sports industry metrics exhibiting double-digit growth. In 2015, both showed extreme imbalance: D of GBAUC = 0.092, D of YRDUC = 0.091. In 2017, both improved to bare coordination: D of GBAUC = 0.600, D of YRDUC = 0.566. In 2019, both reached well coordination: D of GBAUC = 0.851, D of YRDUC = 0.814. By 2021, both achieved quality coordination: D of GBAUC = 0.990, D of YRDUC = 1. This study provides the first evidence from the sports industry that China’s new urbanization model and Document No. 46 are highly effective for synergistic regional economic growth.

## 1. Introduction

In 2014, the Chinese government unveiled the “National New Urbanization Plan (2014–2020)” [1]. Within this comprehensive policy, the first since 1978’s Reform and Opening-up, a noteworthy new development is the clear commitment to a new type of regional development strategy based on the economic dynamism of urban clusters. Given that China’s urbanization rate is significantly lower than that of other industrialized nations and neighboring developing nations, this reform endeavors to address the long-standing issue of unbalanced regional development [2]. In addition, the policy aims to promote investment and innovation by leveraging the urban-driven regional development strategy. Since then, the internal logic of the domestic market and socioeconomic development has been profoundly shifted to a Chinese-style urban clusters economy.

The urban economy is not a new concept. American writer Jane Jacobs first pointed out that the urban economy is the positive externalizations that provided by all the other aspects of the city [3]. China’s urban clusters economy certainly fits this definition. However, the urban economy has never been so densely populated and specialized in human history when China upgraded its development model to an urban clusters economy. Urban clusters in China concentrate not only on people, but also on capital, infrastructure, innovation, and numerous other production factors. Beijing–Tianjin–Hebei urban clusters, Pearl River Delta urban clusters (later upgraded to Greater Bay Area urban clusters, see also below), and Yangtze River Delta urban clusters, which are prioritized in the plan, account for 2.8% of national territory, while these three urban clusters account for 18% of China’s population and 36% of the national GDP in 2013. While China is optimizing the plan experimentally, a few preliminary assessments indicate the plan offers quite advantages over the pre-2014 development model. Evidence from the environmental sector suggests that urban clusters decrease carbon intensity [4]. Urban clusters can also increase the human capital spillover and market integration effects [5]. Meanwhile, it should be acknowledged that the National New Urbanization Plan has been in effect for less than a decade and that there are problems in this development process [6]. For policymakers to fine-tune the plan, it is necessary to gather additional evidence from other sectors regarding the policy’s efficacy.

In 2014, another key national policy was introduced for the sports industry. In October, the State Council issued Document No. 46 [7], which has been regarded as the pivotal moment for China’s sports industry development. Document No. 46 proposes that the sports industry be regarded as an important force for promoting the sustainable development of the economy and society, that the sports industry be deployed to expand domestic demand and promote consumption, and that a five-trillion-CNY sports market be formed by 2025. This is the first document issued by the State Council to promote sports consumption since the founding of New China. Subsequently, a number of national policies supporting the growth of sports consumption have been enacted [8]. In 2019, the State Council called for promoting the sports industry to become a pillar industry of the national economy [9], thereby establishing the sports industry’s indispensable role in the overall economic development.

Urban clusters economy and sports industry are not two independent socioeconomic systems. For instance, Jinjiang, a small county-level city with a population of ~2 million, is China’s major manufacturing hub for athletic footwear. Its highly specialized sports industry has become the economic fulcrum of its city and nearby region [10]. Yao and Liu discussed the relationship between the two systems and their synergistic nature [11]. In brief, the sports industry intensifies market competition, thereby increasing the total output of the sports industry and urban clusters economy. Besides, accelerating the sports industry can foster the growth of the tertiary industry and enhance the regional industrial structure, thereby generating economic growth impetus for the new urbanization. On the other hand, urbanization can provide a conducive environment for the sports industry to expand. Urbanization could boost employment and attract more internal migrant workers, leading to population growth and an increase in sports consumption. In addition, the policy mechanism is an essential guarantee for their coordinated development because it promotes the integration between the sports industry and other industries and accelerates the scale effect in the sports industry.

Despite the abundance of studies on the characteristics, issues, and development paths in the sports industry, there is a dearth of quantitative analyses of the sports industry and regional economy, especially since Document No. 46. Among the few existing literature, Hu and Chen examined the regional sports industry of 17 municipalities in Shandong Province between 2015 and 2016 [12]. They observed that the regions with stronger development of the sports industry radiate to the regions with lower degree of development via human resources, capital, and technology, leading the latter to develop rapidly and subsequently promoting regional economic growth. In another study, Chen and colleagues analyzed China’s sports industry from 2003 to 2017 and noted that, in addition to the well-known regional disparity issue, the level of sports industry development has an overall positive impact on regional economic growth [13]. Using coupling theory, Yao and Liu measured the sports industry agglomeration and regional economic development in 15 Chinese provinces/cities [14]. Their model indicates that the coupling coordination degree has gradually increased between 2010 and 2018. Likewise, Ren and Huang sampled the sports industry and urbanization in Shanghai and concluded that the coupling coordination degree improved between 2014 and 2018 [15].

There are two critical deficiencies in the existing literature. First and foremost, while the existing research examined provincial statistics and drew useful policy recommendations, none accounts for the most recent development model. In 2021, the National People’s Congress endorsed the “Outline of the Fourteen Five-Year Programme for the National Economic and Social Development and the Long-term Goals for 2035 of the People’s Republic of China” (hereafter 14th Five-Year Plan), and the regional development is a key component of the revised development model. Upgrading from the National New Urbanization Plan, the 14th Five-Year Plan identifies a "Two-Horizontal Three-Vertical Urbanization" strategy for urban clusters, under which five urban clusters, including Greater Bay Area urban clusters and Yangtze River Delta urban clusters, are the first to be singled out for upgrading. Therefore, prospective econometrics of China’s development should evaluate the urban clusters economy in place of traditional provincial-level data. Second, to the best of our knowledge, there is no research covering the most recent government statistics regarding the synergistic development of the sports industry and urban clusters economy.

In 2022, during the 20th National Congress, President Xi Jinping emphasized the implementation of high-quality regional development [16]. This reinforces our contention that an updated assessment of the effectiveness of the urban clusters economy since 2014 is of critical importance. Meanwhile, high-quality development of the sports industry is essential for the realization of China’s second Century Goal [17]. Therefore, this study aimed to establish empirical evidence of the coupling coordination between the sports industry and urban clusters economy since the release of Document No. 46 and the National New Urbanization Plan.

## 2. Materials and methods

### 2.1. Sampling

Due to the incomplete information released at the provincial levels, this study examined two of the five top-tier urban clusters outlined in the National New Urbanization Plan and the 14th Five-Year Plan. Greater Bay Area urban clusters are the economic and cultural heart of South China. Because Hong Kong and Macau do not publish sports industry statistics, this study’s sample is limited to the Guangdong province. Yangtze River Delta urban clusters are considered the most economically vibrant center. One of the regions, Anhui Province, only publishes aggregated sports industry statistics and thus cannot be included in the analysis (see index system below). Accordingly, this analysis is based on aggregated statistics from Jiangsu Province, Shanghai Municipality, and Zhejiang Province. The sampling period spans 2015 to 2021, beginning one year after the release of the new policies and ending with the most recent government statistics.

### 2.2. Index system

Coupling coordination analysis is premised on the construction of an objective yet reasonably exhaustive index system, which has a direct influence on the validity of the evaluation outcomes. Typically, a valid evaluation index system should incorporate the weighing method and solve the classification problems inherent in the research question. First, we examined key references in the fields of sports industry and econometrics and established a framework for coupling coordination. In our framework, the sports industry subsystem is divided into three dimensions: industry size, industry quality, and market dynamism [15, 18], whilst the urban clusters economy subsystem is also divided into three dimensions: economy size, economy quality, and consumer dynamism [14].

For the sport industry subsystem: (a) Industry size positively or negatively affects the three dimensions of urban clusters economy. For instance, total output value is explicitly factored into regional GDP calculations. Likewise, industrial agglomeration influences regional GDP and the extent of tertiary industry [11]. (b) Industry quality, such as added value, reflects the development trend of the industry, and its contribution to the regional GDP reflects the high-quality growth of the regional economy [19]. Additionally, added value is a contributor to the GDP per capita [20]. (c) Market dynamism positively or negatively affects the three dimensions of the urban clusters economy. For instance, the manufacture of sporting goods and related products affects the local government’s tax revenue and total retail sales of consumer goods. Emerging as a fast-growing component of tertiary industry, sports services have an impact on personal consumption expenditures as their share of the economy increases [15].

For the urban clusters economy system: (a) Economic size positively or negatively affects the three dimensions of the sports industry. For instance, regional GDP indicates the overall economic outlook, and its scale has a direct impact on business decisions to invest in the development and investment in the relevant industry. Given that the province typically finances the construction of sports facilities in China, local government revenue has a direct impact on the construction of sports facilities. (b) Economic quality positively or negatively affects the three dimensions of the sports industry. Provincial fixed asset investment determines the quality of the basic infrastructure, a prerequisite for the development and expansion of numerous industrial sectors [21]. The added value of tertiary industry is a significant indicator of urbanization, and its size influences various aspects of market dynamism [22]. (c) Consumer dynamism positively or negatively affects the three dimensions of the sports industry. There is a long-term positive relationship between consumer expenditure and the growth of the sports industry [23], necessitating an increase in residents’ income to foster the growth of the sports industry.

Then, considering the industrial activities, key sports sectors, and availability of government statistics, a total of 15 indicators were chosen to construct the evaluation index system, as shown in Table 1. Among the indicators, China sports lottery is an important component listed in the Sports Development Plan [24]. Empirical research has shown that China sports lottery raises funding for the development of regional projects, which can enhance regional economic growth and stimulate residents’ spending [25]. China sports lottery is a useful barometer of the growth of the sports industry. However, sales of the China sports lottery are not included in the annual sports industry statistics but are reported alongside the China Welfare Lottery in the Ministry of Finance’s annual statistics. According to the National Statistical Classification of the Sports Industry [26], China sports lottery falls under the sports service sector. Therefore, we combine the sports service from the annual statistics of the sports industry with the sales of China sports lottery.

**Table 1:** Evaluation index system.

System	Criteria	Indicator	EW (%)	
			GBAUC	YRDUC
Sports industry (S)	S1. Industry size	S1.1. Total output value of regional sports industry	11.13	14.87
		S1.2. Agglomeration of regional sports industry	25.05	11.56
	S2. Industry quality	S2.1. Added value of regional sports industry	10.13	13.29
		S2.2. Proportion of regional sports industry to GDP	9.15	11.09
	S3. Market dynamism	S3.1. Sports service + China sports lottery	13.72	15.14
		S3.2. Manufacture of sporting goods and related products	12.58	19.43
		S3.3. Construction of sports facilities	18.25	14.61
Urban clusters economy (R)	U1. Economic size	U1.1. Regional GDP	13.66	13.61
		U1.2. Local government revenue	11.27	10.51
	U2. Economic quality	U2.1. Fixed asset investment	13.80	11.45
		U2.2. Total retail sales of consumer goods	10.26	12.27
		U2.3. Added value of tertiary industry	13.58	13.04
	U3. Consumer dynamism	U3.1. Regional GDP per capita	11.94	12.66
		U3.2. Disposable income	13.81	13.26
		U3.3. Personal consumption expenditures	11.68	13.19

Note. EW, entropy weight; GBAUC, Greater Bay Area urban clusters; YRDUC, Yangtze River Delta urban clusters.

We used the location quotient to assess the agglomeration of regional sports industry. The location quotient is an index used to measure the spatial distribution of elements in a region, which reflects the level of industrial agglomeration and specialization. The location quotient is calculated as Eq (1):

$$LQ_{ij}=\frac{e_{ij}/e_{i}}{E_{j}/E}$$ (1)

where, *LQ*_*ij*_ denotes the location quotient of sports industry *j* in region *i*; *e*_*ij*_ denotes the added value of sports industry *j* in region *i*; *e*_*i*_ denotes the GDP in region *i*; *E*_*j*_ denotes the added value of the national sports industry; and, *E* denotes the national GDP. If 0 ≤ LQ < 1, the sampled industrial agglomeration lags that of the national average; if LQ = 1, then the sampled industrial agglomeration is the same as the national average; and, if LQ > 1, then the sampled industrial agglomeration leads that of the national average.

The raw data that support the conclusions of this study are available on figshare (DOI: 10.6084/m9.figshare.23921754.v1). Data were extracted from the statistical bulletins published by the National Bureau of Statistics of China and local People’s Governments, including the China Statistical Yearbook, China Statistical Yearbook of the Tertiary Industry, and Statistical Communiqué of the National Economic and Social Development. For the calculation of Yangtze River Delta urban clusters, statistics from Jiangsu Province, Shanghai municipality, and Zhejiang Province were aggregated (e.g., added value) or averaged (e.g., disposable income) using common sense.

### 2.3. Entropy weight method

The entropy weight method is selected for its objectivity and precision to determine the weights of each evaluating indicator. The primary premise of the entropy weight method in the decision-making process is that the significance of an indicator increases as the value difference in the evaluation index system on the same indicator increases, and vice versa. For brevity, readers are referred to Ren and Huang’s work for the detailed procedures [15]. The calculation was performed using the R package creditmodel version 1.3.1. A tailored procedure for this research question is described as follows:

Standardizing the original value is a prerequisite for both the entropy weight method and the coupling coordination degree. Indicators are standardized to dimensionless form as Eq (2):

$$x_{ij}^{\prime }=\frac{xij-\text{min}\;xij}{\text{max}\;xij-\text{min}\;xij}+\text{0}.01,j\in j^{+}$$ (2)

where, $x_{ij}^{\prime }$ denotes the standardized value of the *i*th evaluating system on the *j*th evaluating indicator; *x*_*ij*_ denotes the original value; and, max *x*_*ij*_ and min *x*_*ij*_ denote the maximum and minimum values of the *j*th evaluating indicator, respectively. Since the entropy weight method involves the calculation of natural logarithms, to avoid the meaninglessness of the assigned number, a positive number slightly greater than 0 is often added to the number after standardization of the original value, which is typically set to 0.01 in the literature.

Unique to this study is the objective to evaluate the development status of two distinct regions using standardized values. Greater Bay Area urban clusters and Yangtze River Delta urban clusters had respective populations of approximately 86 million and 227 million in 2022. The socioeconomic magnitude of the two clusters cannot be compared on the same scale. In addition, the structure of their sports industry exhibits distinct characteristics, as Greater Bay Area urban clusters have a relatively balanced structure whilst Yangtze River Delta urban clusters are famous for the specialized manufacture of sporting goods and related products. Should we pool the two regions during standardization, the method penalizes (i.e., influenced by the maximum value) the composite index (see also below) of Greater Bay Area urban clusters and does not reflect the actual industrial structure weight. Therefore, it is reasonable to calculate the standardized values within urban clusters to compare the development status between urban clusters.

### 2.4. Coupling coordination degree model

Coupling coordination originates from physics and refers to the phenomenon whereby two or more systems or forms of motion influence one another. Based on the coupling theory, this study examined the nonlinear interaction between the sports industry and urban clusters economy as the coupling of two systems. The calculation of the coupling coordination degree is as follows:

The composite indexes of the sports industry (*u*_1_) and regional economy (*u*_2_) are calculated as Eq (3):

$$u_{i}=\sum_{j=1}^{n}w_{ij}x_{ij}^{\prime }$$ (3)

where, *w*_ij_ denotes the index weight of the *i*th evaluating system on the *j*th evaluating indicator.

The coupling coordination degree is calculated as Eq (4):

$$\left\{\begin{array}{l} D=\sqrt{\sqrt{\frac{u1\times u2}{{\left(\frac{u1+u2}{2}\right)}^{2}}}\times T}\\ T=\beta 1\times u1+\beta 2\times u2 \end{array}\right.$$ (4)

where, *T* denotes the comprehensive coefficient of coupling coordination degree between the evaluating systems; and, *β*_1_ and *β*_2_ denote the contribution comprehensive coefficients of evaluating systems to be determined. Compared to the regional economy, the regional sports industry is a much smaller sub-economic system, which must be taken into consideration when assigning weights. In light of the government’s plan for the future sports industry to account for 5% of the national GDP, we assign the sports industry 0.05 weight and the regional economy 0.95 weight. The reference level for the coupling coordination degree is provided in Table 2.

**Table 2:** Classification of coupling coordination degree.

Degree	Level	Degree	Level
[0.00, 0.09)	Extreme imbalance	[0.50, 0.59)	Bare coordination
[0.10, 0.19)	Severe imbalance	[0.60, 0.69)	Primary coordination
[0.20, 0.29)	Moderate imbalance	[0.70, 0.79)	Intermediate coordination
[0.30, 0.39)	Mild imbalance	[0.80, 0.89)	Well coordination
[0.40, 0.49)	On the verge of imbalance	[0.90, 1.00]	Quality coordination

Using RStudio version 2023.06.0 Build 421, this study performed spatial visualization of the coupling coordination degree from 2015 to 2021. We extracted the official coordinate system from the National Geomatics Center of China and used AccGIS Pro 3.1.2 to build a customized urban clusters shapefile. After converting the shapefile to geoJSON format, it was imported into R for customized spatial visualization.

## 3. Results

### 3.1. Overview of the two systems

Table 3 provides a summary of the statistics. We calculated the annual average growth rate (AAGR) to highlight the overall development in the seven years sampled. In brief, both the sports industry and urban clusters economy reaped the benefits of Document No. 46 and the National New Urbanization Plan. Five themes merit emphasis here. First, the sports industry developed faster than the urban clusters economy. Between 2015 and 2021, the economies of both urban clusters grew faster than China’s aggregate GDP. The fact that the regional sports industry led the development of urban clusters economies demonstrates its extraordinary growth within the region and the national economic structure as a whole. In the meantime, the lack of change in the industrial agglomeration is not indicative of its stagnation. Given the method of calculation, it could point to the overall rapid growth of other underdeveloped regions. Second, the AAGR of the added value even surpassed that of the total output value, indicating that not only has the sports industry expanded in aggregate but also the quality of its growth has been steadily improving. This is also an important measure of high-quality economic development in urban clusters economies. Third, among the three primary categories of the sports industry, the sports service sector led the way in terms of output growth. This sector, which is valued at over half a trillion CNY in Yangtze River Delta urban clusters, has the highest overall AAGR, which demonstrates its outstanding contribution to the tertiary industry of the urban clusters economy and its strong potential to lead the transition of China’s economic structure. Fourth, statistics on the manufacture of sporting goods and related products indicate that Yangtze River Delta urban clusters have been steadily enhancing their economic uniqueness and value in addition to their existing strength. The double-digit AAGR indicates that the market is far from saturated and could continue to expand in aggregate and high-end values in the future, which could further support the economic strength of the urban clusters economy. Fifth, the extraordinary AAGR in the construction of sports facilities cannot be achieved without fiscal support from the urban clusters economy, a self-validating point demonstrating the coupling coordination between the two systems.

**Table 3:** Overview of the evaluation index system.

Indicator	Greater Bay Area urban clusters								Yangtze River Delta urban clusters							
	2015	2016	2017	2018	2019	2020	2021	AAGR (%)	2015	2016	2017	2018	2019	2020	2021	AAGR (%)
Total output value (100 million CNY)	3208.45	3570.00	3998.03	4912.00	5403.00	5295.44	6258.00	**10.33**	5223.47	5882.69	6695.30	7865.92	9016.06	9279.19	11662.57	**12.45**
Agglomeration of sports industry (%)	1.72	1.70	1.55	1.55	1.53	1.54	1.57	**-1.25**	1.54	1.53	1.43	1.36	1.30	1.41	1.56	**0.36**
Added value of sports industry (100 million CNY)	999.70	1180.00	1321.86	1655.00	1884.00	1811.40	2081.00	**11.45**	1694.38	1996.37	2282.92	2696.06	2975.35	3082.56	3873.49	**12.84**
Proportion of sports industry to GDP (%)	1.37	1.48	1.47	1.70	1.75	1.64	1.67	**3.07**	1.23	1.33	1.36	1.49%	1.48	1.50	1.66	**4.51**
Sports service + China sports lottery (100 million CNY)	996.50	1251.03	1261.23	2541.19	2825.91	2983.34	3535.84	**23.31**	2412.18	2835.14	3157.48	4660.19	5360.37	5107.62	6487.33	**16.26**
Manufacture of sporting goods and related products (100 million CNY)	2350.58	2488.00	2736.80	2423.00	2569.00	2312.10	2698.00	**2.44**	3049.41	3283.28	3537.82	3585.73	3890.00	4171.57	5754.87	**10.06**
Construction of sports facilities (100 million CNY)	13.80	16.00	21.02	195.00	209.00	250.80	241.00	**128.33**	78.66	97.87	112.10	157.51	192.23	210.12	234.31	**17.48**
Regional GDP (100 million CNY)	72813	79512	89879	97278	107671	110761	124370	**8.04**	137967	150037	167803	181472	200139	206033	233095	**7.87**
Local government revenue (100 million CNY)	9365	10390	11315	12103	12651	12922	14103	**6.09**	22097	25621	25114	27444	28236	28526	32304	**5.77**
Fixed asset investment (100 million CNY)	30031	33009	37478	41488	46093	49412	52525	**8.39**	78923	85698	91373	96875	103482	103530	111525	**5.12**
Total retail sales of consumer goods (100 million CNY)	31333	34739	38200	39501	42664	40208	44188	**5.20**	55718	61625	67876	71921	77041	79649	89993	**7.17**
Added value of tertiary industry (100 million CNY)	36956	41446	47488	52751	59773	62541	69147	**9.47**	72346	81515	91232	100772	112505	118294	131650	**9.02**
Regional GDP per capita (CNY)	67503	72787	81089	87096	94172	88200	98285	**5.69**	89580	97466	107949	116270	129510	127340	141224	**6.84**
Disposable income (CNY)	27859	30296	33003	35810	39014	41029	44993	**7.14**	38314	41635	45353	49373	53580	56006	61022	**6.92**
Personal consumption expenditures (CNY)	20976	23448	24820	26054	28995	28492	31589	**6.15**	26486	28372	30113	32610	34776	33352	38999	**5.86**

Note. AAGR, annual average growth rate.

### 3.2. Coupling coordination of the two systems

**Fig 1:**
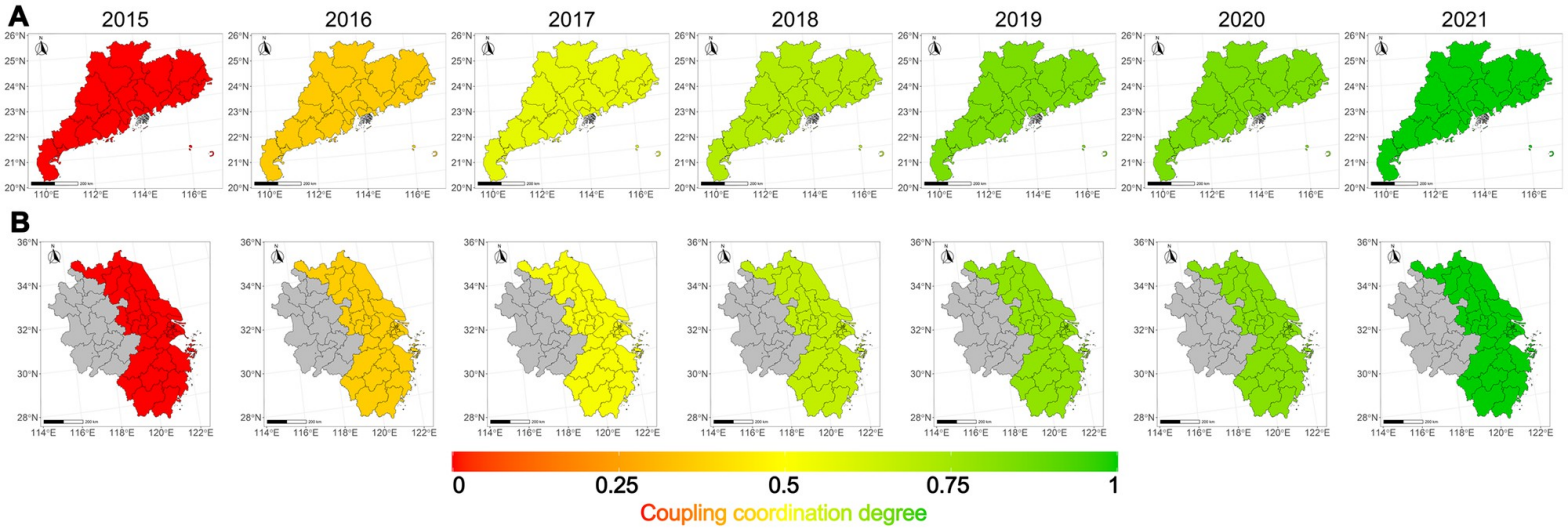
The coupling coordination degree of the sports industry and urban clusters economy from 2015 to 2021: (A) Greater Bay Area urban clusters; (B) Yangtze River Delta urban clusters. The grey color represents a lack of data in the region.

Fig 1 illustrates the progression of the coupling coordination degree in Greater Bay Area urban clusters and Yangtze River Delta urban clusters. In 2015, both regions exhibited an extreme imbalance between the two systems. Over the next two years, the coupling coordination improved to a level of bare coordination, and in the subsequent two years, both regions reached a level of well coordination. Despite the economic drag of COVID-19, both regions achieved quality coordination in 2021.

## 4. Discussion

Promoting coordinated regional development remains one of China’s most urgent socioeconomic challenges [27]. This study centers on the urban clusters as a new economic model and analyzes the coupling coordination of the much smaller but critical sports industry. This is the first unique aspect of this study. The Beijing 2022 Winter Olympics, for instance, were held in Beijing and Zhangjiakou, a prefecture-level city in Hebei province. Should industrial agglomeration (e.g., the winter sports industry) or coupling coordination be measured conventionally, the sampling would not account for the actual scenario or the current national strategy, which prioritizes the growth of urban clusters to foster productivity, innovation, and technological breakthroughs. Therefore, our analyses based on the entropy weight method present an objective assessment of the development status quo of the sports industry and urban clusters economy following the implementation of the National New Urbanization Plan and the revised plan specified in the 14th Five-Year Plan in 2021. This is the first evidence of its kind from the sports industry, and our findings have far-reaching implications for China’s goal to create a new development model in line with the second Century Goal.

It is useful to compare three aspects of the methodologies employed here and in the past. First, the purpose of the entropy weight method is to eliminate human bias when assigning index weight. This necessitates standardized values, which introduce a base value effect when pooling data of interest. Normally, this should not be a concern. In our case, however, the objective evaluation of Guangdong Province would be affected by very large values when aggregating the three economic powerhouses of China. For the calculation of the composite index, we therefore used an independent weighting system. This partially explains the difference between the present study and Yao and Liu’s exhaustive analysis based on provincial-level statistics, in which the coupling coordination between Guangdong’s sports industry and the urban economy was 0.481 in 2015, 0.482 in 2016, 0.490 in 2017, and 0.490 in 2018 [14].

Second, the selection of valid indicators also accounts for the difference between this study and that of Yao and Liu [14]. This is certainly not meant to discredit their methods and results. Instead, we acknowledge the limitation encountered by Yao and Liu when developing their evaluation index system for the sports industry between 2010 and 2018. In 2015, the National Bureau of Statistics of China revised the National Statistical Classification of the Sports Industry [26] in response to Document No. 46 issued by the State Council. Since then, government statistics have provided a much more in-depth picture of the sports industry. Given this limitation at the time, Yao and Liu can only select limited information for their evaluation index system, such as total output value and added value, which reduces its accuracy compared to our evaluation index system, which consists of all categories included in the annual sports industry statistics.

Third, if the evaluation index system is representative, reasonably exhaustive, and valid, the coupling coordination analysis should reveal a similar development status regardless of the indicators chosen. Our study and a previous analysis using Shanghai as a sample confirm this notion. Ren and Huang investigated the coupling coordination between Shanghai’s sports industry and urbanization [15]. To evaluate the two systems, they selected a total of 21 indicators, five of which, including total output value, added value of sports industry, proportion of sports industry to GDP, regional GDP per capita, and disposable income, are used as well in the present analysis. Based on their evaluation index system, the coupling coordination between Shanghai’s sports industry and urbanization was 0.281 in 2015, 0.409 in 2016, 0.515 in 2017, and 0.679 in 2018. Using our evaluation index system to measure the coupling coordination in Shanghai (which can be easily calculated using the deposited raw data), we came to an exact conclusion regarding Shanghai’s sports industry and urban economy: the coupling coordination was 0.093 in 2015, 0.400 in 2016, 0.548 in 2017, and 0.680 in 2018. These three points support the validity of the analysis and results of this study.

At first glance, this study has the inherent limitation that the sampled statistics include both urban and rural regions, and thus do not strictly conform to the definition of an urban clusters economy. Under the grand theme of the future, we consider that this not be regarded as a drawback at all. The "Two-Horizontal Three-Vertical Urbanization" plan specifies that the five key urban clusters will be upgraded first, followed by a second and third batch of urban clusters for expansion and improvement. The long-range objective is for the urbanization rate to reach 73.78% by 2035 [28]. The national policy intends, from a societal perspective, to explore key urban clusters serving as a pilot for rural-urban integration and reduce the income gap between urban and rural populations. Ultimately, to achieve China’s common prosperity, there should be no physical and financial borders separating rural and urban areas.

This study provides the first evidence from the sports industry to support the comprehensive national policy of shifting to a high-quality economy based on urban clusters, and has significant policy implications. Earlier research based on provincial-level panel data from 2005 to 2015 revealed a non-linear relationship between the sports industry agglomeration and economic growth [18]. More recently, Wang and Li examined the effects of policies on the integration of the sports industry and regional economic growth in the Yangtze River Delta and suggested that policies had a two-year lag [29]. This phenomenon has been confirmed in our study. Both sampled regions in 2015 exhibited an extreme imbalance. The coupling coordination then improved at a two-year incremental pace, from barely coordination in 2017 to well coordination in 2019 to quality coordination in 2021.

The strong and swift integration of the sports industry and urban clusters economy is the result of the synergistic amplification of effective government policies during this period. First, the economic agglomeration effect and industrial structure effect constitute key transmission channels through which urbanization shapes industrial development. Greater Bay Area urban clusters and Yangtze River Delta urban clusters lead China’s socioeconomic development with their more advanced industrial structures. National New Urbanization Plan’s issuance paves the way for even more rapid economic acceleration. In 2021, China’s GDP surpasses 114.4 trillion CNY, with a national disposable income of 35128 CNY. Meanwhile, both sampled regions achieved even stronger economic growth, and the disposable income reached 44993 CNY and 61022 CNY for Greater Bay Area urban clusters and Yangtze River Delta urban clusters, respectively. Along with economic progress and the rise in the residents’ standard of living, the demand for a healthy lifestyle and sports consumption has expanded rapidly [19], which is conducive to rapid industrial growth.

Second, the release of Document No. 46 is not only an important milestone for China’s sports industry, but it also creates a new hub for a more sophisticated economic structure and higher-order development spurts. In terms of wealth creation, the profit margins of China’s sports industry are characterized by regional agglomeration, and there are correlations between the profit margins of neighboring provinces [30]. As a result, the accelerated development of the sports industry stimulates synchronized regional growth in neighboring regions. The extraordinary growth of the sports service sector in Greater Bay Area urban clusters and Yangtze River Delta urban clusters demonstrates that the sports industry is indeed reshaping China’s tertiary sector. Particularly, the sports education market should be a robust, predictable sector to support various levels of urban cluster economies, such as the construction of new infrastructure, the creation of new full- and part-time jobs, and the implementation of new technologies [31, 32]. Moreover, as we recently argued, the Chinese path to sports modernization redefines the modernization theory, and the sports industry may usher in a new “Beyond GDP” [33] form of modernization for China and globalization for the world [17]. Ultimately, a highly functioning economy can be superior with a healthy population empowered by the sports industry.

Going forward, China’s sports industry will assume an even greater role in vitalizing high-quality socioeconomic development [17]. In light of this, it is useful to highlight one sub-sector that could continue to expand both the sports industry and urban clusters economy. Our analysis of coupling coordination focuses on China’s leading economic regions, where the sporting goods manufacturing sector accounts for a significant industrial share. Panel data from Yangtze River Delta show that the sporting goods manufacturing sector exhibits a certain degree of crowding out effect [32]. Meanwhile, Chinese brands’ recognition in overseas markets remains irrelevant. In 2021, for example, 98.7% of Li Ning Group’s revenue originated from the domestic market [34]. Therefore, Chinese manufacturers of sporting goods should modify their strategies for international sales, which could support domestic urban clusters economy. The integration of the transcontinental sports industry between China and other nations may also stimulate broader economic growth in other nations [35].

Due to the availability of data, this analysis lacks coverage of the other three top-tier urban clusters defined by the government policy. In the meantime, the current results, in terms of the rapid coupling coordination obtained, may not be applicable to other urban clusters given that each has unique industry structures. The sports industry in Beijing–Tianjin–Hebei urban clusters benefits from the Olympic legacy, and its winter sports-related service sector dominates its high-quality growth. Although Chengdu–Chongqing urban clusters and Middle Reaches of Yangtze River urban clusters lack the industrial base and logistics advantages for developing a comprehensive economic system, these regions have high population density. Future release of consistent government statistics would enable researchers to devise targeted strategies for their coordinated regional development.

Finally, the Chinese experience might be useful for other developing nations seeking to modernize their urbanization and industry structure. The present results indicate that the policy mechanism is an important guarantee for the coordinated industrial development within the broader urban clusters economy, as it realizes the upgrading of economic structure and expedites the formation of the industry’s scale effect. Despite different political structures, modern sports have transcended their competition value to become profoundly ingrained in all aspects of socioeconomic life [36]. Other developing nations could examine China’s policy approaches and devise their tailored policies for fostering comprehensive regional development.

In conclusion, our analysis finds that the sports industry and urban clusters economy in the Greater Bay Area and Yangtze River Delta have attained quality coordination since the 2014 national policy reforms. As China pivots towards high-level urbanization and a leading sports nation, this study offers crucial evidence for the new type of regional development as a kinetic economic engine for China’s overall development agenda on the Chinese path to modernization.
